# Carpal Tunnel Syndrome Assessment with Ultrasonography: Value of Inlet-to-Outlet Median Nerve Area Ratio in Patients versus Healthy Volunteers

**DOI:** 10.1371/journal.pone.0116777

**Published:** 2015-01-24

**Authors:** Tengfei Fu, Manlin Cao, Fang Liu, Jiaan Zhu, Dongmei Ye, Xianxuan Feng, Yiming Xu, Gang Wang, Yuehong Bai

**Affiliations:** 1 Department of Rehabilitation Medicine, Shanghai Jiao Tong University Affiliated Sixth People’s Hospital, Shanghai, China; 2 Department of Ultrasound, Shanghai Jiao Tong University Affiliated Sixth People’s Hospital, Shanghai, China; University of Leicester, UNITED KINGDOM

## Abstract

**Objective:**

To evaluate the diagnostic value of the Inlet-to-outlet median nerve area ratio (IOR) in patients with clinically and electrophysiologically confirmed carpal tunnel syndrome (CTS).

**Methods:**

Forty-six wrists in 46 consecutive patients with clinical and electrodiagnostic evidence of CTS and forty-four wrists in 44 healthy volunteers were examined with ultrasonography. The cross-sectional area (CSA) of the median nerve was measured at the carpal tunnel inlet (the level of scaphoid-pisiform) and outlet (the level of the hook of the hamate), and the IOR was calculated for each wrist. Ultrasonography and electrodiagnostic tests were performed under blinded conditions. Electrodiagnostic testing combined with clinical symptoms were considered to be the gold standard test. Receiver operating characteristic (ROC) curves were used to evaluate the diagnostic value between the inlet CSA and IOR.

**Results:**

The study population included 16 men and 30 women (mean age, 45.3 years; range, 18–83 years). The control population included 18 men and 26 women (mean age, 50.4 years; range, 18–79 years). The mean inlet CSA was 8.7 mm^2^ in healthy controls and 14.6mm^2^ in CTS group (P<0.001). The mean IOR in healthy volunteers (1.0) was smaller than that in patients (1.6, P<0.001). Receiver operating characteristic analysis revealed a diagnostic advantage to using the IOR rather than the inlet CSA (P<0.01). An IOR cutoff value of ≥ 1.3 would yield 93% specificity and 91% sensitivity in the diagnosis of CTS.

**Conclusion:**

The IOR of median nerve area promises to be an effective means in the diagnosis of CTS. A large-scale, randomized controlled trial is required to determine how and when this parameter will be used.

## Introduction

Carpal tunnel syndrome (CTS) is a combination of signs and symptoms due to compression and trapping of the median nerve at the wrist. It is the most commonly reported peripheral nerve entrapment syndrome. A few studies performed in the United States revealed that CTS accounts for 0.2% of all ambulatory care visits [1] and over 500,000 carpal tunnel releases in 2006 [2]. Currently, CTS is typically diagnosed by history and physical examination and electrodiagnostic study results [3,4]. Although this approach is effective for localizing the site of pathology and determining the severity of the condition, electrodiagnostic study has its own limitations: it does not provide information about structures surrounding the nerve, it does not allow visualization of abnormalities intrinsic to the nerve, and it is painful [5].

Over the past years, high-resolution ultrasonography has been proposed as a useful tool for the diagnosis of CTS [6,7]. The attraction of ultrasonography for diagnosis of CTS lies in its wide availability, lower cost, noninvasiveness, and shorter examination time [3]. The measurement of cross-sectional area (CSA) of the median nerve at the wrist is the most widely used ultrasonography method in CTS diagnosis. Normal ranges for median nerve area at the distal wrist crease have varied among reports, ranging from 7.2 to 9.8 mm^2^ [8–11]; the values for diagnosing CTS range from 9 to 15 mm^2^ [12]. The sensitivity and specificity range from 70 to 88% and 57 to 97%, respectively [12]. Much of this variability can be attributed to different study conditions and measurement techniques, along with factors such as age, weight and gender. A single measurement at the wrist could result in a false positive ultrasound diagnosis of CTS such as patients with demyelinating hereditary sensorimotor neuropathy who might have diffuse enlargement of all nerves [13]. Therefore, we hypothesized that if we calculated the ratio of median nerve area at the carpal tunnel inlet as compared to the carpal tunnel outlet (IOR), an IOR measurement would compensate for the interindividual variability in the CSA of the median nerve and yield a more accurate diagnosis of CTS. A ratio would also be less affected by differences in measurement technique [14].

In this study, we aimed to evaluate the diagnostic value of the IOR in patients with clinical and electrodiagnostic evidence of CTS.

## Methods

### Participants

The study was approved by the ethical committee of Shanghai Jiao Tong University Affiliated Sixth People’s Hospital, and all subjects signed written informed consent prior to the investigation. From February to August 2013, patients were recruited by one investigator (T.F.) from the Electromyography Laboratory of Shanghai Jiao Tong University Affiliated Sixth People’s Hospital who had a clinical diagnosis of CTS. Clinical diagnosis of CTS was based on the clinical diagnostic criteria of the American Academy of Neurology [15]. These criteria include: paresthesia; pain; swelling; numbness or weakness of the hand worsened by sleep or sustained hand or arm position; repetitive action of the hand or wrist that is alleviated by shaking the hand or by changing posture; and sensory deficit or atrophy of the thenar eminence muscle. Exclusion criteria were (1) patients < 18 years old and women known to be pregnant; (2) previous carpal tunnel release surgery; (3) prior history of wrist fracture or surgery; (4) clinical evidence of other cervical radiculopathy, polyneuropathy or mononeuropathies.

A control group of healthy volunteers was recruited by the same investigator (T.F.). Only those 18 years of age and older, free of any clinical signs or symptoms of neuropathy, were included. Individuals were excluded if they answered “yes” to any of the following questions: “Do you have carpal tunnel syndrome? Have you ever experienced pain, numbness, tingling or weakness in your hands or arms?”

Demographic factors including age, gender, height and weight were recorded in all participants in the study, and body mass indexes (BMIs) were calculated. We had deposited relevant study files to the Dryad database (http://datadryad.org/review?doi=doi:10.5061/dryad.jt2sm). All subjects in the CTS and control groups underwent electrodiagnostic testing and ultrasonography examinations of both hands.

### Electrodiagnostic testing

All nerve conduction studies were performed at room temperature by a technician (D.Y.). The technician was blinded to clinical status of patients and volunteers. Skin temperature was kept above 33°C. The Keypoint Portable (Alpine BioMed ApS, Skovlunde, Danmark) was used. This nerve conduction study consisted of transcarpal antidromic median and ulnar sensory peak latencies, median conduction velocities, and median distal motor latencies in both upper extremities. These parameters were measured with standard techniques of supramaximal stimulation and surface electrodes. The median and ulnar nerve was stimulated at the wrist and the peak latencies were recorded 14 cm from the ring finger using ring electrodes. The median motor nerve conduction studies were obtained with recording electrodes placed over the abductor pollicis brevis muscle. The active electrode was placed over the muscle belly, and the reference electrode was place over the muscle tendon. A difference of more than 0.4 ms between the median and ulnar sensory peak latencies and/or a median distal motor latency (DML) of more than 4.0 ms was defined as confirmatory electrophysiological evidence of CTS [16].

### Ultrasonography examinations

Ultrasound examinations were performed within 1 week after electrodiagnostic study by a well-trained radiologist (J.Z.) with 10 years of musculoskeletal ultrasonography experience. The radiologist was not permitted to ask the volunteers or patients about symptoms to minimize observer bias. The radiologist was also blinded to the results of clinical examination and electrodiagnostic tests at the time of the ultrasound study. Examinations were performed by using an Aplio 500 Ultrasound System (Toshiba Medical Systems Corporation, Tokyo, Japan) with a 5–13MHz linear array transducer. Subjects were seated with the arm supported to keep a position of wrists and fingers extended, forearm supinated, elbows fully flexed, and shoulder flexed to 60°. Transverse images of the median nerve were obtained at two sites: carpal tunnel inlet (at the level of scaphoid-pisiform) and carpal tunnel outlet (at the level of the hook of the hamate). At each site the CSA of the median nerve was obtained using the trace function by tracing just inside the hyperechoic rim of the nerve. The transducer was kept perpendicular to the nerve to obtain accurate cross-sectional area. No additional force was applied other than the transducer weight to avoid any artificial nerve deformity. The area was recorded at both sites. The inlet-to-outlet median nerve area ratio (IOR), defined as CSA at carpal tunnel inlet/CSA at carpal tunnel outlet, was then calculated. To test inter-rater reliability, two radiologists independently measured the median nerve CSA at the same level on the same day in 20 participants (10 CTS patients and 10 healthy volunteers). Intra-rater reliability was measured in another 20 participants (10 CTS patients and 10 healthy volunteers) who underwent an ultrasound examination, and a second ultrasound test was performed 2 hours after the initial examination by the same radiologist.

### Statistics

Statistics were performed using SPSS 13.0 (Chicago, Illinois, USA). The mean and standard deviation were calculated for each site. The reliability testing was analyzed by means of the intraclass correlation coefficient for continuous measures. The Shapiro-Wilk test was used to confirm the normality of quantitative parameter. An independent sample *t*-test was used to assess gender differences for the CSAs and IOR in the control and CTS group. The same testing was used to compare IOR, inlet CSA, outlet CSA between control group and CTS group. The CSA at each site and IOR were compared to age, height, weight and BMI using partial correlation coefficients, which were also used to confirm the correlation between ultrasound measurements and electrophysiological findings.

To explore the diagnostic value of IOR, 2×2 contingency tables were constructed using 5 different IOR cutoff values, corresponding specificities and sensitivities were calculated (cutoffs of 1.1–1.5 in 0.1 increments). Electrodiagnostic testing combined with clinical symptoms were considered to be the gold standard test. Receiver operating characteristic (ROC) curves were used to compare the diagnostic value between the inlet CSA and IOR, as well as to obtain an optimal value for ultrasound CTS diagnosis. Based on observed specificities and sensitivities, likelihood ratios (LRs) were calculated for positive and negative results (positive and negative LRs) for IOR cutoff values between 1.1 and 1.5. The positive LR indicates how much more likely it is to find an IOR value equal to or larger than a given IOR cutoff in wrists with CTS (sensitivity) as compared with those without (1—specificity). Conversely, the negative LR indicates how much less likely it is to find an IOR value smaller than a given IOR cutoff in wrists with CTS (1—sensitivity) as compared with those without CTS (specificity) [17].

## Results

**Figure 1:**
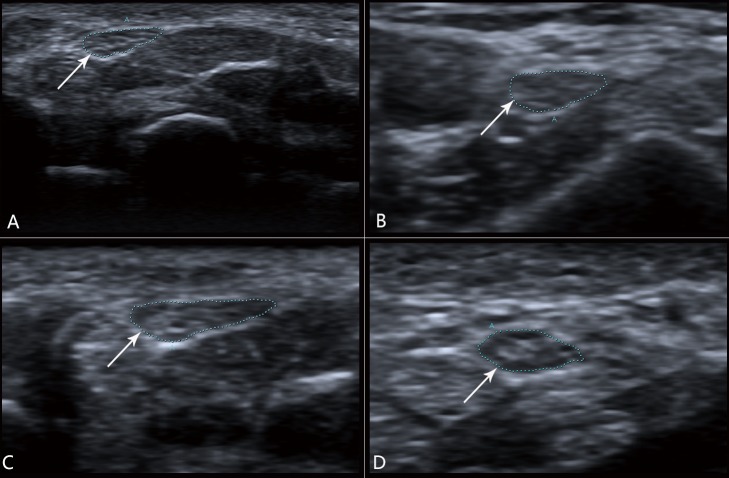
Median nerve images in carpal tunnel inlet (A and C) and carpal tunnel outlet (B and D). The arrow shows the transverse section of median nerve. (A) The normal median nerve at the carpal tunnel inlet measuring 8 mm^2^. (B) The normal median nerve at the carpal tunnel outlet measuring 10 mm^2^. This control had an IOR of 0.8. (C) A patient with clinical and electrodiagnostic evidence of CTS and a median nerve measuring 17 mm^2^ at the carpal tunnel inlet. (D) The median nerve at the carpal tunnel outlet measuring 10 mm^2^. The IOR was 1.7.

Characteristics of the CTS and control groups are outlined in Table 1. Forty-eight patients who were diagnosed with CTS after clinical and electrodiagnostic testing underwent ultrasonography examination. Two patients were excluded from the study because they had bifid median nerves, which resulted in technical difficulty in interpretation of the results. Seven patients had unilateral CTS. In patients with bilateral CTS, the IOR on the least electrodiagnostically affected side was used. In the control group (forty-four volunteers), only the values from the right side were used to avoid artificially lowering the variance. Sample images are provided in Fig. 1. The gender composition, mean age, weight, height, BMI showed no significant difference (*P*>0.05).

**Table 1:** Demographic data of CTS and control groups.

	**CTS group**	**Control group**
Number (n)	46	44
Gender (women/men)	30/16	26/18
Age (years)	45.3±19.8(18–83)	50.4±13.9(18–79)
Weight (kg)	58.2±11.5(42–81)	57.8±11.3(41–77)
Height (m)	1.63±0.08(1.50–1.81)	1.65±0.07(1.52–1.79)
BMI	21.7±3.33(16.2–27.2)	21.1±2.7(16.6–26.2)

All values except for Number and Gender are presented as mean ± standard deviation, followed by range. CTS, carpal tunnel syndrome; BMI, body mass index.

The intraclass correlation coefficients of the inter-rater reliability were 0.88 and 0.79 for the carpal tunnel inlet CSA and outlet CSA. The intraclass correlation coefficients of the intra-rater reliability were 0.93 and 0.85 for the carpal tunnel inlet CSA and outlet CSA. The mean CSA measurements of the median nerve at inlet, outlet and IOR are shown in Table 2. The median nerve CSA at the carpal tunnel inlet in the CTS group was 14.6±4.9 mm^2^ and 8.7±1.2 mm^2^ in the control group with *P*<0.001. At the carpal tunnel outlet, the median nerve CSA was 9.2±2.8 mm^2^ in the CTS group, and 8.8±1.3 mm^2^ in control group (*P* = 0.492). The mean IOR was 1.6±0.3 in the CTS group and 1.0±0.2 in controls (*P*<0.001). There were no statistically significant gender differences between the IOR in the CTS group (*P* = 0.47) or in control group (*P* = 0.75).

**Table 2:** Mean CSA measurements of the median nerve at the carpal tunnel inlet, carpal tunnel outlet, IOR in the CTS and control group.

	**CTS group**	**Control group**	***p***
Inlet	14.6±4.9(8–26)	8.7±1.2(6–12)	<0.001
Outlet	9.2±2.8(5–14)	8.8±1.3(5–11)	0.492
IOR	1.6±0.3(1.2–2.5)	1.0±0.2(0.8–1.4)	<0.001

All values are in mm^2^ except IOR. All values are presented as mean ± standard deviation, followed by range. CSA, cross-sectional area; CTS, carpal tunnel syndrome; IOR, inlet-to-outlet area ratio.

Table 3 shows partial correlation coefficients when the CSA and IOR in control group were compared to age, weight, height, and BMI. Age, weight and BMI correlated significantly with outlet CSA, as well as with inlet CSA except weight. The IOR did not correlate significantly with any of the demographic factors.

**Table 3:** Correlation between median nerve CSAs, IOR and demographic factors in the control group.

	**Age**	**Weight**	**Height**	**BMI**
Inlet CSA	**0.741**	0.178	0.074	**0.388**
Outlet CSA	**0.334**	**0.344**	−0.209	**0.346**
IOR	0.178	−0.236	0.284	−0.114

The correlations between median nerve CSA at each site, IOR (right side only) and age, weight, height, BMI were calculated by partial correlation coefficient. Correlation coefficients with p<0.05 are in indicated bold. CSA, cross-sectional area; IOR, inlet-to-outlet area ratio; BMI, body mass index.

Difference between the median & ulnar sensory peak latencies and DML had a positive correlation with inlet CSA and IOR while a negative correlation with outlet CSA. The median-ulnar latency difference and median nerve DML were 0.72±0.16 ms and 7.02±1.55 ms, respectively. The correlation coefficients between electrophysiological findings and ultrasound measurements are shown in Table 4.

**Table 4:** Correlation between ultrasound measurements and electrophysiological findings in CTS group.

	**Difference**	**DML**
Inlet CSA	**0.779**	**0.763**
Outlet CSA	**−0.630**	**−0.605**
IOR	**0.721**	**0.713**

The median nerve CSA at each site and IOR were compared to difference between the median & ulnar nerve latencies and DML by partial correlation coefficient. Significant values are highlighted with bold (p<0.05). CTS, carpal tunnel syndrome; CSA, cross-sectional area; IOR, inlet-to-outlet area ratio; Difference, difference between the median and ulnar nerve peak latencies; DML, distal motor latency.

**Figure 2:**
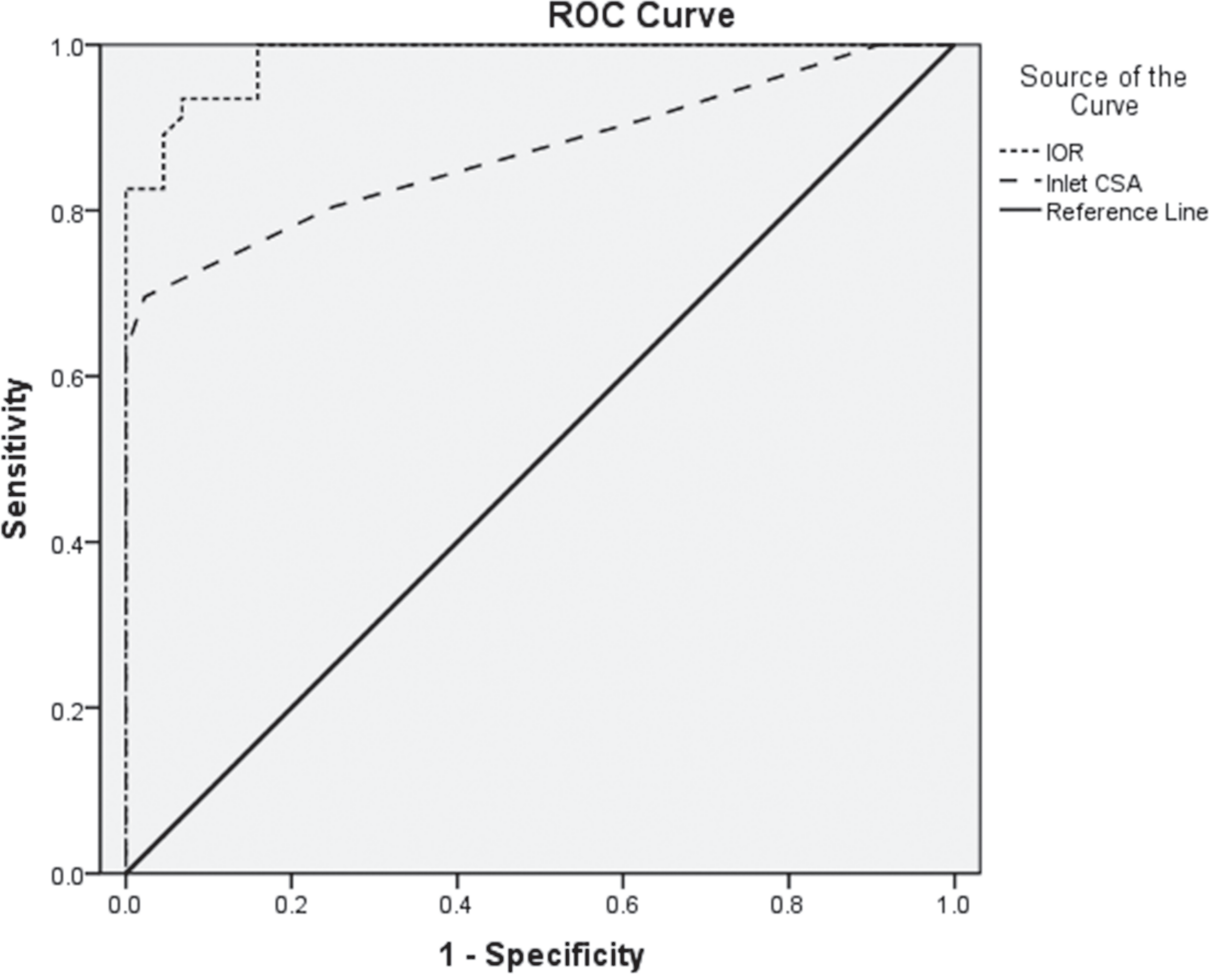
ROC curves for IOR and Inlet CSA. Solid diagonal broken line represents a hypothetical ROC curve from a test that yielded no diagnostic information.

ROC curves were calculated to evaluate specificity and sensitivity of IOR and Inlet CSA in the CTS diagnosis (Fig. 2). The areas under the IOR curve and Inlet CSA curve were 0.98 (95% confidence interval 0.97, 1.00), 0.87 (95% confidence interval 0.79, 0.95), respectively. According to comparisons of the areas under the ROC curves, the discriminating performance of IOR was significantly superior to that of Inlet CSA (*P*<0.01). Table 5 shows that an IOR cutoff value of 1.3 yielded a specificity of 93% and a sensitivity of 91%. An IOR cutoff value of 1.1 yielded a sensitivity of 100% while a specificity of 64%. Conversely, an IOR cutoff value of 1.5 yielded a specificity of 100% but only a sensitivity of 61%. Table 5 also presents LRs for IOR values below (negative LR) and equal to or greater than a specific cutoff value (positive LR).

**Table 5:** Accuracy of IOR according to different IOR cutoff values.

**Cutoff value**	**No. of patients**				**Specificity %**	**Sensitivity %**	**Positive LR**	**Negative LR**
	**FP**	**TN**	**TP**	**FN**				
1.1	16	28	46	0	64	100	2.78	0.00
1.2	7	37	43	3	84	93	5.81	0.08
1.3	3	41	42	4	93	91	13.00	0.10
1.4	2	42	38	8	95	83	16.60	0.18
1.5	0	44	28	18	100	61	Inf.	0.39

Shown were 2×2 contingency tables, calculated specificities and sensitivities, and positive and negative likelihood ratios. IOR values below a given cutoff were considered test negative; values equal to or larger than the cutoff were considered test positive. IOR, inlet-to-outlet area ratio; FP, false-positive; TN, true-negative; TP, true-positive; FN, false-negative; LR, likelihood ratio; Inf., infinity.

## Discussion

The diagnosis of CTS traditionally is based on typical clinical signs and symptoms and may be confirmed by using electrodiagnostic studies [18]. More recently, median nerve sonography has joined the list of potential diagnostic tools [14]. There are a number of criteria used to diagnose CTS by ultrasonography in the literature, such as CSA at the level of the pisiform bone, flattening ratio, swelling ratio, bowing of the flexor retinaculum and median nerve wrist-to-forearm area ratio (WFR) [14,19–21]. Maybe the lack of standard measuring technique is the reason that there is no consensus in the ultrasonographic measurements for CTS diagnosis. The measurement of median nerve CSA at the carpal tunnel inlet was most frequently used for the diagnosis of CTS by ultrasound [12,22]. However, owing to the variability in results and corresponding specificity and sensitivity values, it is difficult to put into use in clinical practice.

In this study, we compared the IOR of median nerve area between patients with clinical and electrodiagnostic evidence of CTS and healthy controls. The IOR difference between the two groups showed strong significance. In addition, the IOR of median nerve area had positive correlations with electrodiagnostic findings. These results indicate that IOR may be useful in the diagnosis of CTS. In order to reveal the diagnostic value of the IOR in CTS diagnosis, ROC curves were used. In the ROC curve analysis, areas under curve (AUC) indicated that IOR (0.98) was more powerful compared to inlet CSA (0.87) in the CTS diagnosis (*P*<0.01). Furthermore, we compared different cutoff values of IOR from 1.1 to 1.5 for purpose of establishing the optimal cut-off value which would balance specificity and sensitivity. Using an IOR cutoff value of ≥ 1.3 to diagnose CTS would yield 93% specificity in healthy controls and 91% sensitivity patients with clinical and electrodiagnostic evidence of CTS in our study. The corresponding positive LR and negative LR were 13.00 to rule in CTS and 0.10 to rule out CTS. Although an IOR cutoff value of 1.1 and 1.5 would yield 100% sensitivity and 100% specificity, it yielded unsatisfactory positive LR and negative LR, respectively. That is to say, an IOR cutoff value of 1.1 or 1.5 would not have enough power to rule in or rule out CTS. Therefore, we considered IOR value of 1.3 as the best cutoff value for the diagnosis of CTS.

Similar to our study, Hobson-Webb LD [14] and Ulaşli [23] also used ratio as a diagnostic measurement for CTS. The former developed a WFR (wrist-to-forearm area ratio) with 100% sensitivity and unknown specificity using area at the distal wrist crease compared to 12cm proximally in the forearm. The latter established a SRmax4 with 93% sensitivity and 98% specificity using the largest median nerve CSA of those measured at carpal tunnel inlet, midtunnel, outlet divided by 4 cm proximal to the wrist crease. Unlike their studies, we developed the IOR using median nerve CSA at the carpal tunnel inlet compared to that at the carpal tunnel outlet. We determined the site of measurements based on a relative position at the carpal tunnel inlet and outlet rather than using absolute values from a single position. Accounting for variations in upper extremity length among subjects, this technique showed similar variation in median nerve CSA with location when it was used in a previous study [7]. Standardized sites for nerve measurements using techniques that account for variation in upper extremity length may improve the precision of median nerve size parameters and should be employed to determine nerve ratio parameters [24].

Recent years, there is a move toward using the largest CSA as the most appropriate measurement for CTS diagnosis. Ziswiler [25] used the largest CSA of median nerve observed between the area proximal to the carpal tunnel inlet and the tunnel outlet, and found a mean value of 12.2 mm^2^ in CTS patients and 7.9 mm^2^ in controls. A cut-off point of 10 mm^2^ yielded a sensitivity of 82%, a specificity of 87%, and an AUC (areas under curve) of 0.89 in their study. Ulaşli [23] revealed that the largest CSA of median nerve was more sensitive in sonographic diagnosis of CTS when the cut-off point was set at 10 mm^2^ (99% sensitivity), however it had a low specificity value (71%), which increased the false positives, as described by the authors. Nonetheless, nerve caliber and swelling is a continuum, and attempts to define single universal threshold values will always have limited success [3]. Furthermore, a recent study [26] proposed that measuring median nerve enlargement at both ends of the carpal tunnel for CTS diagnostics may result in greater diagnostic sensitivity. Therefore, using the CSA of median nerve in carpal tunnel inlet and outlet, we established the inlet-to-outlet area ratio (IOR), which did not correlate significantly with any of the demographic factors, as patients become their own internal controls. Hence, the IOR can help rule out problems of variability between populations when IOR is used to the diagnosis of CTS. This is the primary advantage of using a ratio rather than a single threshold.

An interesting finding was, at the carpal tunnel outlet, the median nerve CSA in CTS group (9.2±2.8 mm^2^) showed no statistical significance (*P* = 0.492) compared to that in control group (8.8±1.3 mm^2^). In contrast, previous studies [19,27,28] found significant differences in Outlet CSA of median nerve between CTS and control group (*P*<0.01). A possible explanation for this discrepancy could be that our study group differed from the population studied from Buchberger [19,27] and Nakamichi [28] with regard to gender composition, age and BMI. MR studies [29] showed the pathologic changes of the median nerve in CTS as nerves swelling at the inlet of carpal tunnel and flattening at the outlet of carpal tunnel. These observations were confirmed with ultrasound [28,30]. Nevertheless, it is unlikely to provide a complete explanation for the differences between our study and previous studies. Ultrasonography is an operator-dependent test and measurements of the median nerve CSA at the carpal tunnel outlet are more technically difficult than the inlet [7]. With appropriate training of operator by adjusting the probe position and ultrasound beam intensity [31,32], this issue can be resolved readily.

This study had several limitations. First, because of the small size of the participants with homogeneous ethnicity and BMI, the generalizability of our results remains unknown. Second, since our study did not include minimal and extreme CTS, the efficacy of the IOR was studied in a relatively small clinical spectrum of CTS patients. However, the focus of this study was to establish the inlet-to-outlet area ratio and then preliminarily verify whether the ratio can be used to diagnose CTS. Third, we used electrodiagnostic testing combined with clinical symptoms as the reference standard in our study. Electrodiagnostic studies have been widely used in the diagnosis of CTS, whereas the false-negative gate has been reported to be between 10% and 15% [16]. Therefore, it is possible that we did not include patients with CTS whose findings were negative at electrodiagnostic tests. Fourth, ultrasound is an operator-dependent examination technique and appropriate experience is required to ensure reliability and reproducibility. In our study, the intraclass correlation coefficients of the inter-rater reliability for the carpal tunnel inlet CSA and outlet CSA were 0.88 and 0.79, respectively. The intraclass correlation coefficient was classified as poor (0.00–0.20), fair (0.21–0.40), good (0.41–0.75), or excellent (>0.75) [33]. Therefore, considering the simple and easily applicable protocol in our study and the satisfactory inter- and intra-rater reliability found in this and other studies [3,8,34], achieving a measure accuracy comparable with ours should be a realistic objective for others. However, the inter- or intra-rater reliability was tested in only 40 of the 90 participants, which also may have been a limitation of this study. Finally, the IOR of median nerve area may be superior to CSA alone in the CTS diagnosis, however, median nerve swelling or involvement at the carpal tunnel outlet will obviously reduce the clinical usefulness of the IOR. In addition, 10%-15% of patients with CTS have an anatomical variation involving a division of the median nerve into two or three parts [35]. Since we considered this variation to be an exclusion criterion, the IOR would not apply to patients with a divided median nerve.

The present study focused on evaluation of CSA measurement of the median nerve for the diagnosis of CTS. Our results demonstrated that the IOR improves the diagnostic accuracy of ultrasound for the diagnosis of CTS. Optimal diagnostic cutoff value was 1.3, resulting in a specificity of 93% and a sensitivity of 91%. Further investigation is needed to determine how and when this parameter will be used.
